# Genetic Predictors of Antipsychotic Efflux Impairment via Blood-Brain Barrier: Role of Transport Proteins

**DOI:** 10.3390/genes14051085

**Published:** 2023-05-15

**Authors:** Regina F. Nasyrova, Natalia A. Shnayder, Sofia M. Osipova, Aiperi K. Khasanova, Ilya S. Efremov, Mustafa Al-Zamil, Marina M. Petrova, Ekaterina A. Narodova, Natalia P. Garganeeva, German A. Shipulin

**Affiliations:** 1 Institute of Personalized Psychiatry and Neurology, V.M. Bekhterev National Medical Research Centre for Psychiatry and Neurology, Saint-Petersburg 192019, Russia; osipova.sm.0@gmail.com; 2 International Centre for Education and Research in Neuropsychiatry, Samara State Medical University, Samara 443016, Russia; 3 Shared Core Facilities “Molecular and Cell Technologies”, V.F. Voino-Yasenetsky Krasnoyarsk State Medical University, Krasnoyarsk 660022, Russia; stk99@yandex.ru (M.M.P.); katya_n2001@mail.ru (E.A.N.); 4 Department of Psychiatry, Russian Medical Academy for Continual Professional Education, Moscow 125993, Russia; abdyrahmanova_peri@mail.ru; 5 Department of Psychiatry and Addiction, Bashkir State Medical University, Ufa 450008, Russia; efremovilya102@gmail.com; 6 Department of Physiotherapy, Faculty of Continuing Medical Education, Peoples’ Friendship University of Russia, Moscow 117198, Russia; alzamil@mail.ru; 7 Department of General Medical Practice and Outpatient Therapy, Siberian State Medical University, Tomsk 634050, Russia; garganeeva@gmail.com; 8 Centre for Strategic Planning and Management of Biomedical Health Risks Management, Moscow 119121, Russia; shipgerman@gmail.com

**Keywords:** antipsychotic, transport protein, P-gp, BCRP, MRP1, adverse drug reaction, pharmacogenetics, ABCB1, ABCG2, ABCC1, single nucleotide variant, personalized psychiatry, efflux, pharmacogenetic testing

## Abstract

Antipsychotic (AP)—induced adverse drug reactions (ADRs) are a current problem of biological and clinical psychiatry. Despite the development of new generations of APs, the problem of AP-induced ADRs has not been solved and continues to be actively studied. One of the important mechanisms for the development of AP-induced ADRs is a genetically-determined impairment of AP efflux across the blood-brain barrier (BBB). We present a narrative review of publications in databases (PubMed, Springer, Scopus, Web of Science E-Library) and online resources: The Human Protein Atlas; GeneCards: The Human Gene Database; US National Library of Medicine; SNPedia; OMIM Online Mendelian Inheritance in Man; The PharmGKB. The role of 15 transport proteins involved in the efflux of drugs and other xenobiotics across cell membranes (P-gp, TAP1, TAP2, MDR3, BSEP, MRP1, MRP2, MRP3, MRP4, MRP5, MRP6, MRP7, MRP8, MRP9, BCRP) was analyzed. The important role of three transporter proteins (P-gp, BCRP, MRP1) in the efflux of APs through the BBB was shown, as well as the association of the functional activity and expression of these transport proteins with low-functional and non-functional single nucleotide variants (SNVs)/polymorphisms of the *ABCB1*, *ABCG2*, *ABCC1* genes, encoding these transport proteins, respectively, in patients with schizophrenia spectrum disorders (SSDs). The authors propose a new pharmacogenetic panel “Transporter protein (PT)—Antipsychotic (AP) Pharmacogenetic test (PGx)” (PTAP-PGx), which allows the evaluation of the cumulative contribution of the studied genetic biomarkers of the impairment of AP efflux through the BBB. The authors also propose a riskometer for PTAP-PGx and a decision-making algorithm for psychiatrists. Conclusions: Understanding the role of the transportation of impaired APs across the BBB and the use of genetic biomarkers for its disruption may make it possible to reduce the frequency and severity of AP-induced ADRs, since this risk can be partially modified by the personalized selection of APs and their dosing rates, taking into account the genetic predisposition of the patient with SSD.

## 1. Introduction

**Figure 1:**
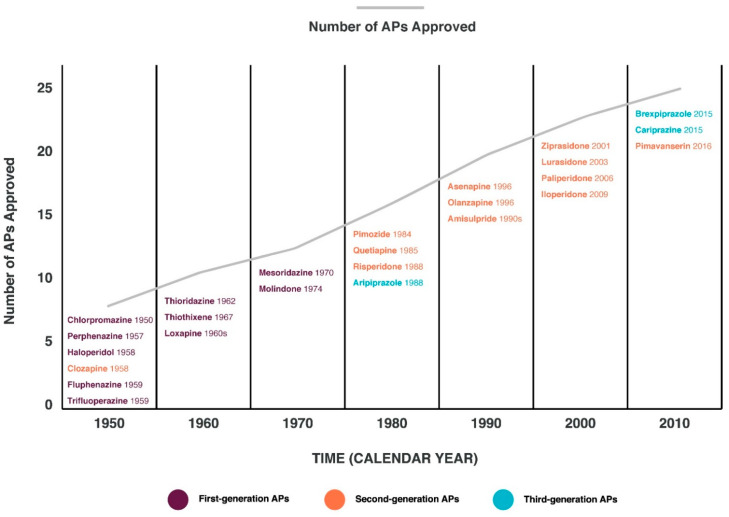
Timeline of antipsychotics (APs) [12].

Schizophrenia spectrum disorders (SSDs) are one of the serious and socially significant mental disorders, the prevalence of which reaches millions of cases in the world [1,2]. For the treatment of SSDs, psychotropic drugs of the antipsychotics (APs) group are widely used [3]. Despite the generation of new APs (Figure 1), the problem of AP-induced adverse drug reactions (ADRs) has not been solved [4]. The accumulated experience of predicting and purposeful prevention of ADRs caused by AP indicates that most of them can be prevented (for example, neurotoxic effects of AP) and/or significantly reduce the frequency and severity of symptoms of these ADRs (for example, cognitive disorders, psychoses, extrapyramidal disorders, etc.) [5,6,7]. This problem leads to: a decrease in the quality of life of patients; a decrease in adherence of patients with SSDs to chronic APs therapy; the development of SSDs’ pseudo-resistance to the prescribed APs; progression of SSDs [8,9]. The study of the mechanisms of development of AP-induced ADRs is based on changes in their metabolism and transport, depending on the following risk factors for ADRs [8,10,11]: (1) modifiable factors (choice of APs, dosage, dosing regimen, consideration of comorbid conditions, etc.) [8,10]; (2) non-modifiable factors (gender, age of patients, genetic predisposition) [8,10].

The study of genetic predisposition to the development of AP-induced neurotoxic ADRs is based on associative genetic studies and genome-wide studies of single nucleotide variants (SNVs) or polymorphisms of the candidate genes encoding key enzymes and proteins involved in the metabolism, transport, cumulation, excretion of APs and their active metabolites [8,10,11]. An important aspect of the efficacy and safety of SSD therapy, especially the prediction and prevention of the development of neurotoxic ADRs, is a genetically determined impairment of the efflux of APs across the blood-brain barrier (BBB) [13,14,15,16].

The purpose of our narrative review is to substantiate the need to develop a new pharmacogenetic panel and decision-making algorithm for assessing the cumulative risk of developing AP-induced neurotoxic ADRs in patients with SSDs, taking into account genetically determined changes in the functional activity and/or expression level of transporter proteins involved in the efflux of APs through the BBB.

## 2. Blood-Brain Barrier

**Figure 2:**
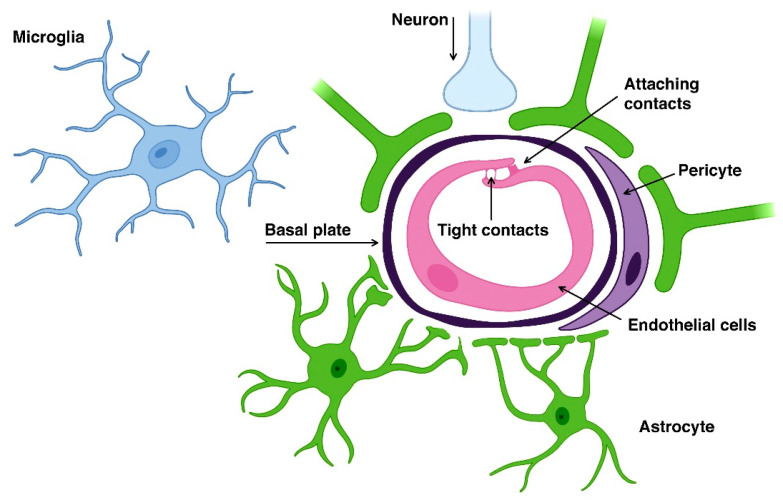
Neurovascular unit of the blood-brain barrier.

The BBB is a complex heterogeneous brain system with several levels of selective transport, regulation, and protection, capable of maintaining central nervous system (CNS) homeostasis and protecting the brain from potentially harmful endogenous and exogenous substances [17,18,19]. The BBB is a physical and metabolic barrier between the brain and the systemic circulation that serves to regulate and protect the brain microenvironment [20]. The structural units that make up the BBB perform not only protective, but also regulatory, nutritional and excretory functions. The main functional and anatomical elements of the BBB are brain capillary endotheliocytes, astrocytes, neurons, and pericytes, which are a “neurovascular unit” [21] (Figure 2).

The BBB consists of a monolayer of endothelial cells in the capillaries of the brain [18,19,22]. The limited coverage of the BBB by the brain occurs due to the presence of tight contacts (occlusal zones) between neighboring endothelial cells and a relatively small number of fenestra and pinocytic vesicles in the endothelium of cerebral arterioles, capillaries, and venules [23]. The endothelial cells of the brain capillaries are surrounded by pericytes and astrocyte pedicels. Due to the presence of the BBB, circulating molecules do not have free access to the brain [18,19,22].

## 3. Transcellular Drug Transport via the Blood-Brain Barrier

Transcellular transport of biologically active substances via the BBB can be carried out in the following ways [21,24]:

1. simple diffusion;
2. facilitated diffusion;
3. endocytosis via receptor-mediated transcytosis;
4. efflux transport.

### 3.1. Simple Diffusion

Simple diffusion through the BBB is carried out in the direction of the concentration gradient. It is a spontaneous process that depends on the magnitude of the concentration gradient [21,23,25]. A necessary condition for it is the high lipophilicity of the transported substance. Fat-soluble molecules can cross the cell membrane and passively diffuse via the BBB from blood to the brain [21,25]. There is a correlation between increased lipid solubility and the rate and extent of their entry into the brain. In general, the more fat-soluble a molecule or drug is, the more easily it will penetrate the brain tissue. Thus, inorganic molecules O_2_, CO_2_, H_2_O quickly penetrate the BBB. Also, some drugs, such as anesthetics and heroin, enter the brain in this way [21,24].

### 3.2. Facilitated Diffusion

Facilitated diffusion through the BBB, mediated by transport proteins, also occurs in the direction of the concentration gradient [23,25]. However, molecules that are not able to penetrate the membrane of endotheliocytes on their own bind to carrier proteins and are transported through endocytosis. Solute transporters (SLCs) are a superfamily of membrane transport proteins that facilitate the bidirectional movement of solutes across the cell membrane [23,25,26,27]. Polar molecules can be transported through the membrane of BBB endothelial cells [27,28]. Unlike their ATP-binding cassette (ABC) transporter counterpart, SLC transport does not require ATP, as it is driven either by electrochemical gradients (i.e., Na+ or H+ gradient) or by concentration gradients established by transported solutes [26,28]. Therefore, SLCs are classified as either lightweight transporters or secondary active transporters. Generally, SLC mediates the entry into the brain of essential nutrients such as glucose, amino acids, nucleosides, monocarboxylates, organic anions and cations, as well as the efflux of certain metabolites. Some drugs (including L-DOPA and others) are also transported to the brain by these transporters [21,24].

### 3.3. Endocytosis via Receptor-Mediated Transcytosis

Endocytosis via receptor-mediated transcytosis (PMT) is the movement of molecules and ions across the cell membrane against any of the gradients (electrochemical, osmotic, etc.) using the energy of ATP [29,30,31,32]. Receptor-mediated transcytosis is a class of transport systems that use the BBB endothelial-cell vesicular transport system to transport substrates into the brain. Usually, PMT is induced by the binding of large molecules such as peptides and proteins to receptors (for example, insulin receptor, transferrin receptor, low density lipoprotein receptor and related protein, etc.) that are highly expressed on the endothelial cell membrane [29,30]. Receptor-mediated endocytosis is commonly used by viruses to penetrate the plasma membrane. When the pH of the endosome drops, the viral proteins change their configuration, which allows them to exit the endosome. Typically, nutrients such as iron, insulin, and leptin are transported to the brain via such an endocytic event, also known as transcytosis [21,24]. Molecules for drug transcytosis (conjugates with ligand and drugs via linkers or nanoparticles) are being developed. Cell-penetrating peptides, receptor-targeted peptides and monoclonal antibodies can be used as ligands. Ligand binding to a receptor on the surface of endothelial cells induces endocytosis. Exosomes containing conjugates are being studied. They move in the cytoplasm and merge with the opposite plasma membrane, releasing them. Then, transcytosed drugs saturated with conjugates, or released drugs from conjugates, exert their activity on the neurons of the central nervous system. However, clinical trials of drugs for the treatment of SSDs are difficult, although this strategy based on transcytosis is a promising method of transporting AP to the brain.

### 3.4. Efflux Transport

The term “antipsychotic efflux” refers to the process of their active removal from nerve cells (neurons) and the brain as a whole using a protein pump embedded in the cell membrane, neurons and endotheliocytes of the BBB. At the same time, the direction of the efflux of APs is their transport from the brain to the blood with the participation of transport proteins [21].

In recent years, much more attention has been paid to studies of this pathway of transcellular transport across the BBB [33,34]. The most important transport efflux mechanism is believed to be carrier-mediated excretion of APs from the brain to blood. BBB endothelial cells contain numerous membrane transporters involved in the influx or efflux of various major substrates such as electrolytes, nucleosides, amino acids, and glucose [27,33,35]. Efflux transport is based on the so-called ATP-Binding Cassette (ABC) transport proteins associated with ATP [27,35,36]. ABC transport proteins have an affinity for a broad category of solutes, especially for large fat-soluble molecules with a number of nitrogen and oxygen atoms in their structure. These ABC transport proteins use ATP hydrolysis to pump molecules across the membrane and hence they can cause solute efflux against a concentration gradient [13,35,36]. P-glycoprotein (P-gp: ABCB1) and breast-cancer-associated protein (BCRP: ABCG2) are the main transporters of ABC efflux in the BBB [34,37,38,39,40].

Transport proteins of the ABC family for the efflux of APs across the BBB are increasingly recognized as important determinants of the distribution of APs in the CNS and their excretion from the brain [27,41]. The P-gp as transport protein in many cases has shown itself to be a key element of the BBB, which can actively transport a huge number of lipophilic drugs from the endothelial cells of the brain capillaries that form the BBB. In addition to P-gp, other transporter proteins, such as members of the multidrug resistance protein (MRP) family and BCRP, appear to contribute to the efflux of APs across the BBB [20].

The implications of all these transport proteins at BBB level include the minimization or prevention of AP-induced neurotoxic ADRs [13,35,36,41] and aggravating SSDs [11,36] or development of pseudoresistance to APs [11,13]. On the other hand, ABC transport proteins may also limit the central distribution of APs used to treat SSDs, increasing the risk of developing therapeutic resistance [13,36,39].

Therefore, knowledge of genetically-determined changes in the functional activity and expression of the aforementioned BBB transport proteins can help form a new personalized strategy for predicting the elimination of APs from the brain and provide new therapeutic opportunities for therapeutically resistant SSDs.

## 4. Transport Proteins of Antipsychotic Efflux via the Blood-Brain Barrier

The human ABC vector superfamily contains 49 members, which are subdivided into 7 subfamilies (from ABCA to ABCG) [39,42]. These transport proteins are localized on various membranes of cell organelles (with the exception of ABCE and ABCF), where they function as ATP-dependent and unidirectionally pump various endogenous and exogenous compounds transmembrane [35,42]. As outflow pumps, these transport proteins perform a wide range of physiological functions, including protective, excretory, and regulatory functions. They create barriers between the systemic circulation and many organs, such as the brain, cerebrospinal fluid (CSF), placenta, and testes, thereby limiting the penetration of APs and toxic metabolites, ensuring their active efflux (excretion) and, therefore, protecting these organs [36]. They are also expressed in the liver and kidneys, where they secrete xenobiotics and endogenous compounds. In addition, they limit the absorption of APs into the systemic circulation due to the release of APs into the gastrointestinal tract. Transport proteins of the ABCB family regulate many endogenous molecules that affect lipid and bile acid synthesis, antigen presentation, heme and iron homeostasis, transport and homeostasis of steroid hormones, and signaling molecules [42]. Currently, 15 transport proteins involved in the transport of APs through cell membranes and tissue barriers are the most studied (Table 1).

**Figure 3:**
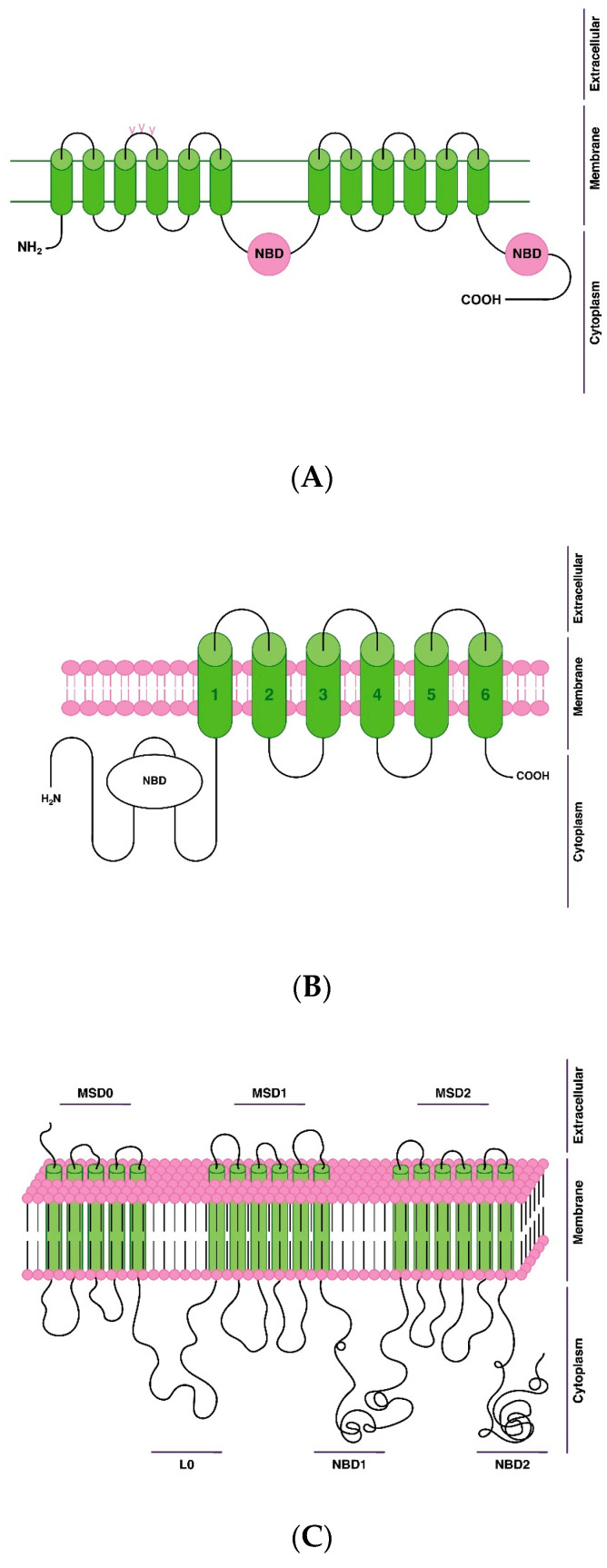
Scheme of the structure and localization of antipsychotic efflux transport proteins via blood-brain barrier on the cell membrane of endotheliocytes of the blood-brain barrier: (**A**)—P-glycoprotein (P-gp); (**B**)—breast cancer resistance protein (BCRP); (**C**)—multidrug resistance-associated protein 1 (MRP1). Note: L0—ligand 0; nucleotide binding domain—NBD; membrane spanning domain—MSD.

The most studied and clinically significant transport proteins that provide the efflux of AP across the BBB and the membrane of target neurons of AP action [39,46] (Table 2, Figure 3):

- P-gp, or multidrug resistance protein 1 (MDR1);
- breast cancer resistance protein (BCRP);
- multidrug resistance-associated protein (MRP11).

Glycoprotein-P (P-gp) is a membrane transport protein with a wide range of endogenous and exogenous substrates. P-gp is located in hepatocytes, enterocytes and epithelial cells of the proximal renal tubules, neurons, and endotheliocytes of histo-hematic barriers, including the BBB. Increased activity of P-gp is associated with the development of drug-resistant forms of SSD, and reduced activity is associated with delayed AP efflux through the BBB and with the risk of the development of neurotoxic ADRs [13,36,47].

P-gp (or MDR1) is a member of the ATP-binding cassette (ABC) transporter superfamily and is an ATP-dependent efflux pump for drugs and other xenobiotics. ABC proteins transport various molecules across extracellular and intracellular membranes. The ABC family proteins are divided into seven subfamilies (ABC1, MDR/TAP, MRP, ALD, OABP, GCN20, White). This protein is a member of the MDR/TAP subfamily and is encoded by the *ABCB1* gene. Members of the MDR/TAP subfamily are involved in multidrug resistance [36,42].

The secondary structure of the P-gp protein is well known. This transport protein has a single polypeptide chain with N- and C-ends located inside the cytoplasmic region, and 12 transmembrane domains are located inside the plasma membrane. Also, P-gp contains 2 nucleotide binding domains (NBDs) that act as ATP binding sites. The first extracellular loop of P-gp contains three glycosylation sites [48].

P-gp expression in the brain is highest at the level of the frontal, medial and mediobasal cortex, in the hippocampus, tail, and also in other organs: adrenal glands, liver, gallbladder, small and large intestines, kidneys, ovaries, and fallopian tubes (in women) [45].

P-gp is encoded by the *ABCB1* gene (chr7: 87,503,017-87,713,323 (GRCh38/hg38)). The molecular structure of the *ABCB1* gene includes 28 exons and 28 introns [44]. The *ABCB1* gene is highly polymorphic. The literature mentions about 100 SNVs/polymorphisms identified in different regions. However, only some of them are associated with the efflux of APs and lead to clinically significant changes in their transport [47,49,50,51]. The identification of low-functional and non-functional SNVs/polymorphisms of the *ABCB1* gene is of clinical interest, since it is associated with an increased risk of AP-induced neurotoxic ADRs and a decreased safety of short-term and, especially, chronic psychopharmacotherapy.

BCRP is a membrane protein, ATP-binding cassette transporter. It is encoded by the *ABCG2* gene. BCRP is one of five members of the human ABC protein superfamily G subfamily, along with ABCG1, ABCG4, ABCG5, and ABCG8. All members of the subfamily are semi-transporters and thus are thought to function as homo- or heterodimers or perhaps even as larger oligomeric structures [52].

Another feature of the ABC transporters G subfamily is that the nucleotide binding domain (NBD) is N-terminal to the transmembrane domain, while the opposite is true for other subfamilies of ABC transporters [38].

All members of the ABCG family, with the exception of BCRP, are known to be lipid transporters. In contrast, BCRP demonstrates perhaps the broadest substrate specificity and transports hydrophobic compounds, cations, anions, and drug conjugates [53]. It is known that this protein is involved in the transportation of APs and may play a role in the development of multidrug therapeutic resistance [37,38,52,53,54]. This protein consists of 655 amino acid residues, and contains one glycosylation site, one intramolecular disulfide bond and one intermolecular one. The intermolecular S–S bond provides stability of this transport protein dimer [38]. Basically, it is located on the cell membrane as a dimer, but can form oligomers up to a homododecamer (12 subunits) [52,54].

The BCRP expression in the brain is highest at the level of the frontal, medial and mediobasal cortex, in the hippocampus, tail, and also in other organs: seminal vesicles, testicles (in men), small and large intestines, placenta, lungs, thyroid gland, adrenal glands, myocardium [45].

The BCRP is encoded by the *ABCG2* gene (chr4:88,090,150-88,231,628 (GRCh38/hg38)). The human *ABCG2* gene consists of 16 exons and 15 introns [44]. At least 38 SNVs of the *ABCG2* gene are mentioned in the literature, and in some sources 45–50 SNVs/polymorphisms of this gene [49,50,51].

The MRP1 is a protein associated with multidrug resistance 1, is encoded by the *ABCC1* gene and is a member of the ABC superfamily [55]. It transports various molecules across extracellular and intracellular membranes. This transport protein is a member of the MRP subfamily that is associated with multidrug resistance. MRP1 is a peculiar member of the ABC transporter superfamily in several aspects. This transport protein has an unusually wide substrate specificity and is able to transport not only a wide range of neutral hydrophobic compounds, but also promote the efflux of numerous glutathione, glucuronate, and sulfate conjugates [55,56]. The transport mechanism of MRP1 is also complex: the composite substrate binding site provides both cooperativity and competition between different substrates [56]. This versatility and ubiquitous distribution in tissues make this transporter suitable for participation in various physiological functions, including the transport of various drugs, including APs [55].

This protein consists of five main domains (membrane spanning domains (MSD0, MSD1, MSD2)) and nucleotide-binding domains (NBD1 and NBD2) [56,57].

MRP1 expression in the brain is highest at the level of the frontal, orbitofrontal, and medial cortex, as well as in other organs: small and large intestines, liver, ovaries, fallopian tubes and endometrium (in women), adipose tissue, tonsils, lungs, pancreas, skin, bone marrow [45].

MRP1 is encoded by the *ABCC1* gene (chr16:15,949,138-16,143,257 (GRCh38/hg38)). The *ABCC1* gene contains 31 exons [44]. In studies of various populations, especially those of Asian and European origin, a large number of SNVs/polymorphisms of the *ABCC1* gene have been found. Studies have shown that only a few of the 71 discovered SNVs/gene polymorphisms changed the amino acid sequence of the transport protein they encode, so *ABCC1* is considered a highly conserved gene. Most of the genetic variations that have been described are found in intron sequences rather than exons [49,50,51].

## 5. Genetic Predisposition to Reduction of Antipsychotic Efflux via the Brain-Blood Barrier

Genetic variants influence the effects of APs in terms of favorable and unfavorable results of psychopharmacological therapy, the development of ADRs, and the neurotoxicity of APs [58,59]. Many studies have shown that pharmacokinetics and neurotoxic ADRs induced by APs vary in patients with SSDs with certain genetic profiles. There is sufficient evidence of a clinically significant effect of patient genotype on the balance between benefit and risk of a wide range of first and new generation APs. Evidence-based guidelines with pharmacotherapeutic recommendations for combinations of specific APs and genotypes or predicted phenotypes in patients with SSDs are needed to implement the acquired knowledge of pharmacogenetics into daily clinical psychiatric practice [9,60].

In the case of a genetically determined decrease in the functional activity or expression of the P-gp, BCRP, and MRP1 transport proteins at the level of BBB endothelial cell membranes, the efflux of APs from the brain into the blood is disturbed to varying degrees (decreases significantly, insignificantly, or moderately) [39]. This, in turn, leads to an increase in the time of exposure of these APs to the brain, an increased risk of cumulation during chronic (long-term) psychopharmacotherapy, and an increased risk of developing serious AP-induced neurotoxic ADRs [36]. The accumulation of APs ultimately leads to a slowdown in their metabolism due to the enzymatic system, and therefore the phenotype of such patients with SSDs is more often referred to as a slow metabolizer rather than a slow transporter [10].

### 5.1. Phenotyping of Patients Depending on Antipsychotic Efflux Reduction via the Blood-Brain Barrier

Pharmacogenetic testing (PGx) has become a more popular laboratory diagnostic method over the past decade, but it is not yet a routine test in psychiatry. However, there is growing evidence that a significant proportion of neurotoxic ADRs in patients with SSDs are affected by a phenotype determined by homozygous or heterozygous carriage of full functional, low functional and non-functional allelic variants of the genes encoding transport proteins responsible for efflux of the first and new generations APs through the BBB [13,27,38,61].

There are several organizations such as the Dutch Pharmacogenetics Working Group (DPWG)), the Clinical Pharmacogenetics Implementation Consortium (CPIC) [62], the Canadian Drug Safety Pharmacogenomics Network (CPNDS) [63], the French National Network of Pharmacogenetics (Réseau) (RNPGx) [64], and the Russian Society of Pharmacogenetics, Pharmacokinetics and Personalized Therapy (SPPPT) [65], who develop clinical guidelines to help the clinician translate the results of previously performed associative genetic and genome-wide associative (GWAS) studies into clinical guidelines for treatment. In general, these organizations recommend pharmacogenetic testing in routine patient care if the clinical benefit to patients is considered significant, such as by reducing the risk of ADRs or the risk of drug treatment failure (therapeutic resistance or pseudo-resistance). However, most international and national clinical guidelines on clinical pharmacogenetics do not take into account the potential effect of SNVs/polymorphisms of the genes encoding transport proteins on the efflux of APs from the brain into the blood via the BBB, the strength of the interaction, or the complex interaction caused by the combination of SNVs/polymorphisms of the genes encoding the P-gp, BCRP, and MRP1 transport proteins and enzymes involved in the APs metabolism, when there are multiple biotransformation pathways. Not surprisingly, these recommendations sometimes contradict each other [66].

Additional sources of information about the pharmacogenetics of APs are the Summary Product Characteristics (SmPC) approved by the European Medicines Agency (EMA) [67] and other agencies, as well as instructions for prescribing APs approved by the US Federal Drug Agency (FDA) [68]. The number of APs with pharmacogenetic information in their SMPC or labels has increased over the years due to regulatory guidelines and policies. In addition, the PharmGKB website [69] is an open support tool for the pharmacogenetics of AP with collected and classified evidence for combinations of APs and other drugs and the genes.

As we better understand the influence of various genetic variants on the pharmacokinetics and levels of APs in the brain, possible personalized approaches for prediction and prevention of neurotoxic ADRs of AP therapy for SSDs become more and more clear.

Depending on the decrease in the functional activity of the key transport proteins that carry out the speed of the efflux of APs via the BBB from the brain into the blood [37,38,39], three phenotypes of patients with SSDs can be distinguished: poor transporter; intermediate transporter; extensive transporter.

#### 5.1.1. Poor Transporter

Poor transporters (PT, also known as “poor metabolizers”—PM [70,71]) are patients whose transport proteins have a significantly reduced functional activity, resulting in a significantly reduced efflux of APs via the BBB. The PT phenotype occurs when both alleles of the *ABCB1, ABCG2* and/or *ABCC1* genes carry non-functional SNVs and give rise to the synthesis of transport protein with impaired functional activity or significantly reduce its expression in the BBB.

Typically, SSDs patients having this phenotype are homozygous for non-functional or low-functional SNVs/polymorphisms of the gene(es) *ABCB1, ABCG2* and/or *ABCC1* encoding one or more transport proteins (P-gp, BCRP, and/or MRP1, respectively). Accordingly, in such patients, APs accumulate in the brain in high concentrations, which leads to a significant increase in the risk of developing serious AP-induced neurotoxic ADRs.

#### 5.1.2. Intermediate Transporter

Intermediate transporters (IT, also known as “intermediate metabolizers”—IM [70,71]) are patients whose transport proteins have a moderately reduced functional activity, resulting in a moderately reduced efflux of APs via the BBB. Typically, SSDs patients with this phenotype are heterozygous for non-functional or low-functional SNVs/polymorphisms of the gene (es) *ABCB1, ABCG2* and/or *ABCC1* encoding transport proteins (P-gp, BCRP, and/or MRP1, respectively). In such patients, the synthesis of a “defective” transport protein or a moderate decrease in the expression of a normal transport protein at the level of the BBB occurs, as a result of which the functional activity of the transport protein and the efflux of APs efflux via the BBB are reduced. Accordingly, with intermediate transporters, APs accumulate in the brain, which leads to a moderate increase in the risk of AP-induced neurotoxic ADRs.

#### 5.1.3. Extensive Transporter

Extensive transporters (ET, also known as “extensive metabolizers”—EM [70,71]) are patients whose transport proteins have normal functional activity, which ensures a preserved (average) rate of efflux of APs via the BBB. SSDs patients with this transport protein phenotype are homozygous for a fully functional (“wildtype”) SNVs/polymorphisms of the genes) *ABCB1, ABCG2* and/or *ABCC1* encoding transport proteins (P-gp, BCRP, and/or MRP1, respectively). Accordingly, in these patients with SSDs, the risk of developing AP-induced neurotoxic ADRs is population average (mild).

### 5.2. Prediction of a Genetically Determined Decrease of Antipsychotic Efflux via the Blood Brain Barrier

The tactics of a psychiatrist who prescribes APs to patients with SSDs need a personal approach [7,37,38,61]. Thus, in carriers of risk alleles of non-functional or low-functional SNVs/polymorphisms of the *ABCB1, ABCG2* and/or *ABCC1* genes encoding transport proteins involved in the efflux of APs via the BBB, it is important to cumulatively evaluate as many previously studied non-functional and low-functional SNVs/polymorphisms of the genes encoding transport proteins involved in efflux through the BBB as possible, for APs of the first and new generations. Such a cumulative assessment of the genetic risk of speed decrease of the efflux of APs via the BBB and the development of AP-induced neurotoxic ADRs significantly modifies and significantly improves the currently existing personalized psychopharmacotherapy strategy for SSDs and explains the importance of developing PGx and its application in real clinical psychiatric practice [12].

In this regard, it is possible to conditionally divide the SNVs/polymorphisms included in this narrative review into groups: low-functional and non-functional SNVs/polymorphisms of the *ABCB1* gene; low-functional and non-functional SNVs/polymorphisms of the *ABCG2* gene; low-functional and non-functional SNVs/polymorphisms of the *ABCC1*; proven in experimental (on an animal model) and clinical (in patients with mental disorders) studies, the effects of risk alleles of non-functional and low-functional SNVs/polymorphisms of these genes on changes in the activity of the transport proteins encoded by them and the level of their expression in the BBB in patients with SSDs.

To search and update our knowledge, information in the following open access databases may be used: (1) databases for evaluating the expression of transport proteins and their encoding genes (The Human Protein Atlas [45], The Human Gene Database (GeneCards [44], and databases “Gene” and “Protein” of the US National Library of Medicine [50]; (2) database of SNVs/polymorphisms of human genes (SNPedia) [49], Online Mendelian Inheritance in Man (OMIM) [43], SNP database of the US National Library of Medicine [50]; (3) pharmacogenetic database (PharmGKB) [51].

## 6. Antipsychotics-Substrates of Transport Proteins

The results of the role of transport proteins in the efflux of APs via the BBB conducted in the framework of this review are summarized and presented in Table 3.

Table 3 shows that SNVs/polymorphisms of the *ABCB1* gene are of the greatest clinical interest in psychiatric practice, since the P-gp encoded by this gene is involved in the efflux through the BBB of many APs. The least studied is the *ABCC1* gene, whose role in PGx continues to be actively studied. On the other hand, some APs (e.g., clozapine, olanzapine, quetiapine, etc.) are cleared through the BBB by several transporter proteins, which is consider important when clinically interpreting the results of PGx in real psychiatric practice. This demonstrates that a comprehensive approach to assessing the contribution of the SNVs/polymorphisms of these genes that affect the efflux of APs of the first and new generations is relevant and scientifically substantiated (Table 4).

## 7. Discussion

The use of PGx to choose therapy for SSDs is increasing [72,73,74], and the implementation of recommendations based on the pharmacogenomics of APs may improve the treatment outcomes of patients with treatment-resistant SSDs [71,75]. Numerous studies have shown improved response rates to APs associated with the use of pharmacogenomic testing in clinical settings [71,72]. However, genetic biomarkers of the disturbance of the efflux of APs across the BBB are not yet included in known PGx. A good example of a system for assessing the genetic contribution to the pharmacokinetics and pharmacodynamics of APs, used in foreign psychiatric practice, is the GeneSight Psychotropic algorithm developed by a group of scientists based at the Mason Clinic (USA) [76]. The test is non-invasive and easy to use: only a buccal epithelium scraping is required to collect a patient’s DNA sample. The results are provided to the psychiatrist or patient within 36 h. The GeneSight panel is based on a multi-gene multivariate genetic test that takes into account the characteristics of the genotype and phenotype of a particular patient, as well as information on drug metabolism. The analysis is performed on 59 allelic variants of 8 genes (*CYP1A2, CYP2C9, CYP2C19, CYP3A4, CYP2B6, CYP2D6, HTR2A* and *SLC6A4*). The attending physician is provided with information already analyzed by the program based on the results of PGx testing of a patient with a mental disorder. In its conclusion, GeneSight contains a list of APs and antidepressants divided into 3 categories: “use as directed”; “use with caution”; “use with increased caution and more frequent monitoring.” In addition, the conclusion contains additional information that helps the psychiatrist decide whether to prescribe or cancel drugs in a particular patient. Thus, this PGx algorithm allows the psychiatrist to make a decision on the appointment of psychopharmacotherapy without the involvement of a clinical pharmacologist in most cases [77,78]. The disadvantages of this method are: the inability to study SNVs/polymorphisms of the genes encoding APs transport proteins; the absence of the cumulative effect of the studied SNVs/polymorphisms and assessment of several genes encoding APs transport proteins; the inability to fully characterize the genetically determined changes in the functional activity of this transport protein(s) and the level of its (their) expression in the BBB.

Another pharmacogenetic test, Genecept Assay, allows psychiatrists to easily make a decision on the appointment of APs and antidepressants. The Genecept Assay results analysis covers a wide range of drugs used for the psychopharmacotherapy of mental disorders, including depression, obsessive-compulsive disorders, schizophrenia, attention deficit hyperactivity disorder, bipolar disorder, etc. The study is conducted on allelic variants in 20 genes, including *5HT2C, MC4R, DRD2, COMT*, and the genes encoding key liver cytochrome P450 isoenzymes. The results of DNA testing are provided to the patient and their attending physician within 8 working days, including recommendations for the selection and dosing of APs. This may help to increase the effectiveness and safety of psychopharmacotherapy [78].The disadvantages of this method are: the absence in the test of SNVs/polymorphisms of the genes encoding APs transport proteins; the impossibility of assessing the risk of decrease in the transport of APs through the membrane of neurons and the BBB; the impossibility of assessing genetically determined changes in the functional activity of the APs transport proteins and the level of their expression at the level of the BBB and endothelial cell membrane of target neurons of the action of APs.

We have formulated a hypothesis that a new pharmacogenetic approach based on the genotyping of alleles of low-functional and non-functional SNVs/polymorphisms of the *ABCB1, ABCG2, ABCC1* genes and phenotyping of patients with SSDs (ET, IT and PT) will allow the development of a new personalized algorithm for assessing the cumulative risk of impaired expression and functional activity of transport proteins and the efflux of APs through the BBB and, on a scientific basis, to develop a riskometer for the development of AP-induced neurotoxic ADRs, as well as a decision-making system for psychiatrists.

### 7.1. Perspective Pharmacogenetic Panel

We presented a new panel of PGx (Table 4), based on the search for low-functional and non-functional SNVs/polymorphisms of the *ABCB1, ABCG2, ABCC1* genes in patients with SSDs.

This PGx for assessing the cumulative genetic risk of changes in the efflux of APs from brain to blood via the BBB, based on the microarray method—a new pharmacogenetic panel “Transport proteins (PT)—Antipsychotic (AP)—Pharmacogenetic test (PGx)” or “PTAP-PGx”, takes into account the characteristics of the genotype and phenotype of patients with SSDs, and also provides important information about the genetically determined decrease in the efflux of APs from brain to blood via the BBB.

According to the results of this PGx, patients with SSDs are divided into 3 phenotypes (ET, IT and PT) by testing 25 low-functional and non-functional SNVs/polymorphisms of the *ABCB1, ABCG2, ABCC1* genes. This PGx is designed to assess the cumulative risk of developing AP-induced neurotoxic ADRs associated with a pronounced reduction of the efflux of APs of the first and new generation via the BBB through three transport proteins (P-gp, BCRP, and MRP1). The psychiatrist is provided with already-analyzed information on the results of PGx. The conclusion contains a list of APs, divided into 4 categories: “use as directed” (homozygous carriage of functional (wild-type) allelic variant of the *ABCB1, ABCG2, ABCC1* genes); “use with caution” (heterozygous carriage of low-functioning allelic variants of the *ABCB1, ABCG2, ABCC1* genes); “use with increased caution and with more frequent monitoring” (homozygous carriage of low-functioning allelic variants of the *ABCB1*, *ABCG2*, *ABCC1* genes); “do not use” (homozygous carriage of non-functional allelic variants of the *ABCB1, ABCG2, ABCC1* genes).

### 7.2. Riskometer for PTAP-PGx

To ensure the convenience and speed of clinical interpretation of the results of PTAP-PGx for practicing psychiatrists, we additionally developed a riskometer for AP-induced neurotoxic ADRs, taking into account the genetically determined impairment of the efflux of APs from brain to blood via the BBB.

The riskometer includes the following criteria for evaluating PGx results: qualitative characteristics of changes in the functional activity of the transporter protein—poor/intermediate/extensive transporter; quantitative characterization (through a change in protein expression)—reduced/normal expression of a given transport protein.

**Figure 4:**
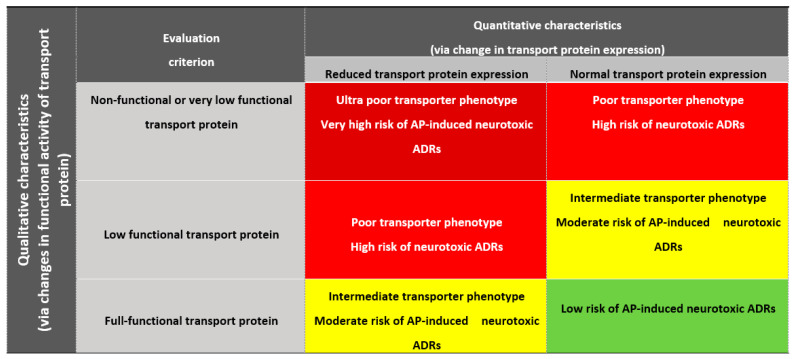
Riskometer for PTAP-PGx. Note: ADRs—adverse drug reactions; AP—antipsychotic; dark red and red zones—efflux of AP via the blood-brain barrier (BBB) is significantly reduced; yellow zone—efflux of AP via the BBB is moderately reduced; green zone—efflux of AP via the BBB is normal.

The cumulative risk of a decrease in the efflux of APs via the BBB, the development of AP-induced neurotoxic ADRs, and a decrease in the effect of APs can be easily assessed by the attending physician based on the data presented in Figure 4 from negligible (average population “green zone”) to very high (“red zone”).

The frequency of use of therapeutic drug monitoring (TDM) of APs in the blood [79] varies across countries. We propose a differentiated approach to the choice of the frequency of TDM in patients with SSDs depending on the risk group for developing neurotoxic ADRs, which is presented in the PTAP-PGx Decision Algorithm.

### 7.3. PTAP-PGx Decision Algorithm

The riskometer for the PTAP-PGx panel has been supplemented by the authors with a decision-making algorithm for a practicing psychiatrist.

#### 7.3.1. Very High Risk of Antipsychotic-Induced Neurotoxic Adverse Drug Reactions

- Cancellation of the previously used AP, transported from brain to blood via the BBB mainly by this transport protein, if the patient is already taking this AP.
- Refusal to prescribe an AP transported from brain to blood via the BBB mainly by this transport protein, if the patient has not previously taken this AP.

#### 7.3.2. High Risk of Antipsychotic-Induced Neurotoxic Adverse Drug Reactions

- Reduction by ~50% of single and daily doses of a previously used AP, transported mainly by this transport protein, if the patient is already taking this AP.
- The start of pharmacotherapy with a decrease of ~50% from the average starting dose of a newly prescribed AP, transported from brain to blood via the BBB mainly by this transport protein, if the patient has not previously taken this AP.
- A very slow pace of increasing the dose of AP (reducing the rate of increasing the dose by 2 times: for example, 1 time in 4 weeks instead of 1 time in 2 weeks).
- Clinical monitoring of possible neurotoxic ADRs is recommended in monotherapy with these APs and, especially, in polytherapy with APs.
- TDM of the level of this AP in the blood or plasma at least once every 3 months.
- Refusal of polytherapy with the appointment of two or more APs, transported from brain to blood via the BBB mainly by this transport protein.
- With low efficiency of monotherapy with this AP, transported mainly by this transport protein, it is possible to additionally prescribe an AP, transported mainly by another transport protein.

#### 7.3.3. Moderate Risk of Antipsychotic-Induced Neurotoxic Adverse Drug Reactions

- Reduction by ~25% of single and daily doses of a previously used AP, transported mainly by this transport protein, if the patient is already taking this AP.
- The start of pharmacotherapy with a decrease of ~25% from the average starting dose of a newly prescribed AP, transported mainly from brain to blood via the BBB by this transport protein, if the patient has not previously taken this AP.
- Slow rate of increase in the dose of this AP (decrease in the rate of increase in the dose of AP by ~25%: for example, once every 3 weeks instead of once every 2 weeks).
- Clinical monitoring of possible neurotoxic ADRs in AP polytherapy is recommended.
- TDM of the level of this AP in the blood or plasma once every 6 months.

#### 7.3.4. No Risk (or Minimal Risk) of Antipsychotic-Induced Neurotoxic Adverse Drug Reactions

- It is possible to prescribe the average or maximum allowable (according to the current instructions for this AP), according to indications, therapeutic single and daily doses of the previously used AP, transported mainly by this transport protein, if the patient is already taking this AP.
- Start of pharmacotherapy with an average starting dose of a newly prescribed AP, transported mainly by this protein, if the patient has not previously taken this AP.
- The average rate of increasing the dose of AP (according to the current instructions for this AP).
- Dynamic observation is recommended during long-term use of this AP in monotherapy, or when it is prescribed in high (or maximum allowable) doses, or in polytherapy with APs transported from brain to blood via the BBB with the participation of this transport protein.
- TDM of the blood (or plasma) level of this AP and/or its active metabolites according to indications (when prescribing this AP in high or maximum allowable doses or in polytherapy) or TDM of the level of this AP in the blood or plasma at least once every 12 months.

## 8. Limitations

We recognize that to identify the PT phenotype, it is not enough to detect a single non-functional variant of one of the proposed genes, since the violation of the outflow of APs is a multifactorial and polygenic condition. In this regard, it is assumed that the new panel proposed by the authors will include all or the maximum number of options listed in Table 4. In addition, it is interesting to study the role of possible haplotypes in variants of selected genes associated with changes in the rate of efflux of APs through the BBB. We believe that the proposed panel of polymorphisms can complement information about other relevant genes in relation to the pharmacogenetics of the first and new generations of APs. The PTAP-PGx panel will be useful as an addition to existing or new panels that allow you to assess the genetic predisposition to slow down APs metabolism.

We did not include in this narrative review highly functional SNVs/polymorphisms of the *ABCB1*, *ABCG2*, *ABCC1* genes associated with an increase in the efflux of APs through the BBB. This is due to the fact that the authors summarized information on the genetic predisposition to the development of AP-induced neurotoxic ADRs, which is associated with a decrease in the efflux of APs via the BBB. On the contrary, highly functional allelic variants of the *ABCB1*, *ABCG2*, *ABCC1* genes may be associated with an increase in the efflux of APs via the BBB, a decrease in response to APs, and the development of therapeutically resistant SSDs. Undoubtedly, highly functional SNVs of the candidate genes responsible for increasing the efflux of APs via the BBB and increasing the risk of therapeutic resistance are important from a clinical point of view.

Research is ongoing, and we hope to prepare a new review on this topic in the near future.

## 9. Conclusions

There is no doubt that there may be pharmacogenetic differences in patients with SSDs seeking treatments that potentially affect the increase in the level (cumulation) of APs in the brain. This contributes to a decrease in the effectiveness of APs and an increase in the burden of neurotoxic ADRs with an additional potential risk of serious complications (psychosis, severe cognitive disorders, severe extrapyramidal disorders, etc.). Evidence for the use of pharmacogenomically-targeted therapy in SSDs is still evolving. At the same time, special attention is paid to the safety of APs, the pseudoresistance of SSDs to AP therapy, the burden of neurotoxic ADRs, and patient adherence to treatment. Further research is needed as scientific and logistical barriers still exist before we can expect widespread implementation of clinical pharmacogenetics focused on the study of brain-to-blood efflux of APs into actual clinical practice. However, pharmacogenetics as an interdisciplinary science has great potential for individualizing the medical treatment of SSDs.

The development of personalized biomedicine poses new challenges for us, among which the safety of chronic psychopharmacotherapy of SSDs, and the prediction and prevention of AP-induced neurotoxic ADRs, are among the highest priorities. PTAP-PGx may become a new “key” to unlock the mysteries of cases of paradoxical worsening of SSDs during acute and, especially, chronic psychopharmacotherapy.

## Figures and Tables

**Table 1:** Transporter proteins and their encoding genes.

Gene: OMIM *	Chromosomal Location **	Protein **	Amino Acid Content ***
*ABCB1:* 171050	chr7: 87,503,017-87,713,323 (GRCh38/hg38)	Multidrug Resistance Protein 1 (MDR1) P-Glycoprotein 1 (P-gp)	1280
*ABCB2:* 170260	chr6: 32,845,209-32,853,816 (GRCh38/hg38)	Transporter 1 (TAP1)	808
*ABCB3:* 170261	chr6: 32,821,831-32,838,770 (GRCh38/hg38)	Transporter 2 (TAP2)	653
*ABCB4:* 171060	chr7: 87,398,988-87,480,435 (GRCh38/hg38)	Multidrug Resistance Protein 3 (MDR2) P-Glycoprotein 3	1279
*ABCB11:* 603201	chr2: 168,915,468-169,031,396 (GRCh38/hg38)	Bile Salt Export Pump (BSEP)	1321
*ABCC1:* 158343	chr16: 15,949,138-16,143,257 (GRCh38/hg38)	Multidrug Resistance-Associated Protein 1 (MRP1)	1531
*ABCC2:* 601107	chr10: 99,782,602-99,853,741 (GRCh38/hg38)	Multidrug Resistance-Associated Protein 2 (MRP2)	1545
*ABCC3:* 604323	chr17: 50,634,777-50,692,253 (GRCh38/hg38)	Multidrug Resistance-Associated Protein 3 (MRP3)	1527
*ABCC4:* 605250	chr13: 95,019,835-95,301,475 (GRCh38/hg38)	Multidrug Resistance-Associated Protein 4 (MRP4)	1325
*ABCC5:* 605251	chr3: 183,919,934-184,018,010 (GRCh38/hg38)	Multidrug Resistance-Associated Protein 5 (MRP5)	1437
*ABCC6:* 603234	chr16: 16,149,565-16,223,617 (GRCh38/hg38)	Multidrug Resistance-Associated Protein 6 (MRP6)	1503
*ABCC10:* 612509	chr6: 43,427,366-43,451,994 (GRCh38/hg38)	Multidrug Resistance-Associated Protein 7 (MRP7)	1464
*ABCC11:* 607040	chr16: 48,164,842-48,249,973 (GRCh38/hg38)	Multidrug Resistance-Associated Protein 8 (MRP8)	1382
*ABCC12:* 607041	chr16: 48,080,882-48,156,018 (GRCh38/hg38)	Multidrug Resistance-Associated Protein 9 (MRP9)	1359
*ABCG2:* 603756	chr4: 88,090,150-88,231,628 (GRCh38/hg38)	Breast Cancer Resistance Protein (BCRP)	655

Note: * from the open database OMIM Online Mendelian Inheritance in Man [43] ** from the open database GeneCards: The Human Gene Database [44] *** from the open database The Human Protein Atlas [45].

**Table 2:** Substrates and inhibitors of ABC transport proteins expressed in endotheliocytes of the blood-brain barrier.

Transport Protein	Substrates	Inhibitors
P-glycoprotein (P-gp)	Amisulpride Aripiprazole Azenapine Chlorpromazine Chlorprothixene Clozapine Fluphenazine Flupentixol Olanzapine Paliperidone Periciazine Quetiapine Risperidone Sertindole Sulpiride Trifluoperazine Ziprasidone Zuclopenthixol	Amiodarone Atorvastatin Azithromycin Bromocriptine Captopril Carvedilol Chlorpromazine Clarithromycin Cyclosporine Diltiazem Dipyridamole Erythromycin Fluoxetine Hydrocortisone Itraconazole Ketoconazole Loratadine Nifedipine Propafenone Propranolol Quinidine Ranolazine Reserpine Ritonavir Sertraline Simvastatin Spironolactone Ticagrelor Verapamil Vinblastine Vincristine Warfarin
Breast Cancer Resistance Protein (BCRP)	Aripiprazole Chlorpromazine Clozapine Haloperidol Olanzapine Paliperidone Quetiapine Risperidone Sulpiride	Boceprevir Cyclosporine Dipyridamole Fluconazole Gefitinib Itraconazole Imatinib Ketoconazole Nicardipine Nifedipine Omeprazole Pantoprazole Reserpine Ritonavir Tamoxifen Telaprevir
Multidrug Resistance-Associated Protein 1 (MRP1)	Clozapine	
		Clotrimazole Cyclosporine Disulfiram Flavonoids Verapamil

**Table 3:** Antipsychotics—Substrates of Transport Proteins.

P-gp	BCRP	MRP1
Amisulpride Aripiprazole Azenapine Chlorpromazine Chlorprothixene Clozapine Fluphenazine Flupentixol Olanzapine Paliperidone Periciazine Quetiapine Risperidone Sertindole Sulpiride Trifluoperazine Ziprasidone Zuclopenthixol	Aripiprazole Chlorpromazine Clozapine Haloperidol Olanzapine Paliperidone Quetiapine Risperidone Sulpiride	Clozapine

Note: P-gp—P-glycoprotein; BCRP—Breast Cancer Resistance Protein; MRP1—Multidrug Resistance-Associated Protein 1.

**Table 4:** Low functioning and non-functional single-nucleotide variants of the genes encoding antipsychotics transport proteins.

*ABCB1*	*ABCG2*	*ABCC1*
rs1045642 rs1128503 rs2032582 rs2235048	rs2231142 rs2231137 rs72552713 rs3116448 rs758900849 rs192169063 rs34264773 rs199753603 rs12721643 rs41282401 rs1061017 rs1061018 rs3201997 rs750568956 rs753759474 rs752626614 rs372192400 rs200894058 rs199854112 rs769734146 rs34783571 rs45605536 rs58818712 rs750568956 rs2622604	rs212090

Note: ABCB1—ATP Binding Cassette Subfamily B Member 1, ABCG2—ATP Binding Cassette Subfamily G Member 2, ABCC1—ATP Binding Cassette Subfamily C Member 1.

## Data Availability

Not applicable.
